# Insights into the biology of the rat lungworm, *Angiostrongylus cantonensis*

**DOI:** 10.1186/s13071-025-06790-3

**Published:** 2025-04-30

**Authors:** Chasen D. Griffin, Vanessa O. Ezenwa, Robert H. Cowie

**Affiliations:** 1 Pacific Biosciences Research Center, University of Hawaii at Manoa, Honolulu, HI USA; 2 Department of Ecology and Evolutionary Biology, Yale University, New Haven, CT USA; 3 Odum School of Ecology, University of Georgia, Athens, GA USA; 4 Department of Infectious Diseases, College of Veterinary Medicine, University of Georgia, Athens, GA USA

**Keywords:** *Angiostrongyluscantonensis*, Rat lungworm, Angiostrongyliasis, Helminth, Eosinophilic meningitis, Zoonosis, Parasitic disease

## Abstract

*Angiostrongylus cantonensis* is a parasitic nematode with a complex life cycle involving rats as definitive hosts and gastropods as intermediate hosts. The parasite can infect other organisms, including humans, in which it causes neuroangiostrongyliasis, a globally emerging but neglected disease. This primer reviews the biology of *A. cantonensis* including its life cycle and development in its natural, accidental, and paratenic hosts, as well as its expanding geographic distribution. It then considers recent advances in *A. cantonensis* research followed by exploring areas that are ripe for further investigation into this fascinating parasite.

**Graphical Abstract:**

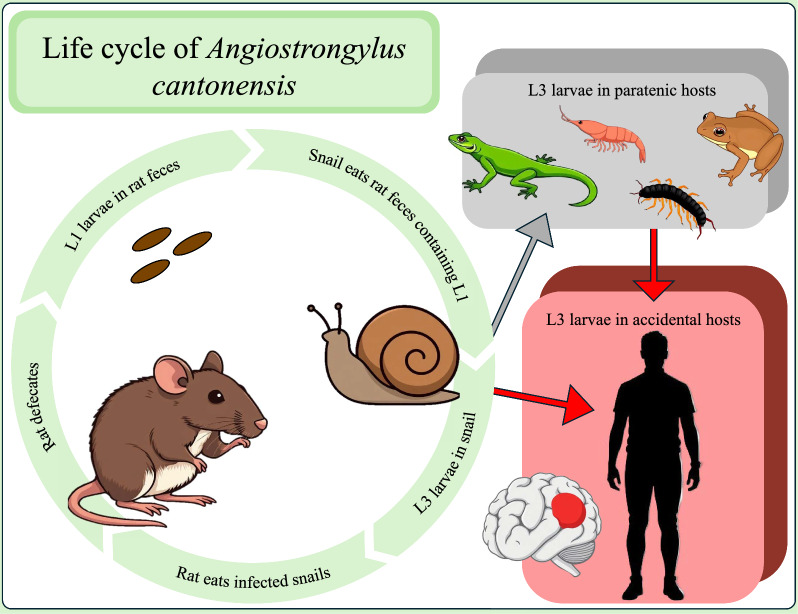

## What is *Angiostrongylus cantonensis*?

**Fig. 1:**
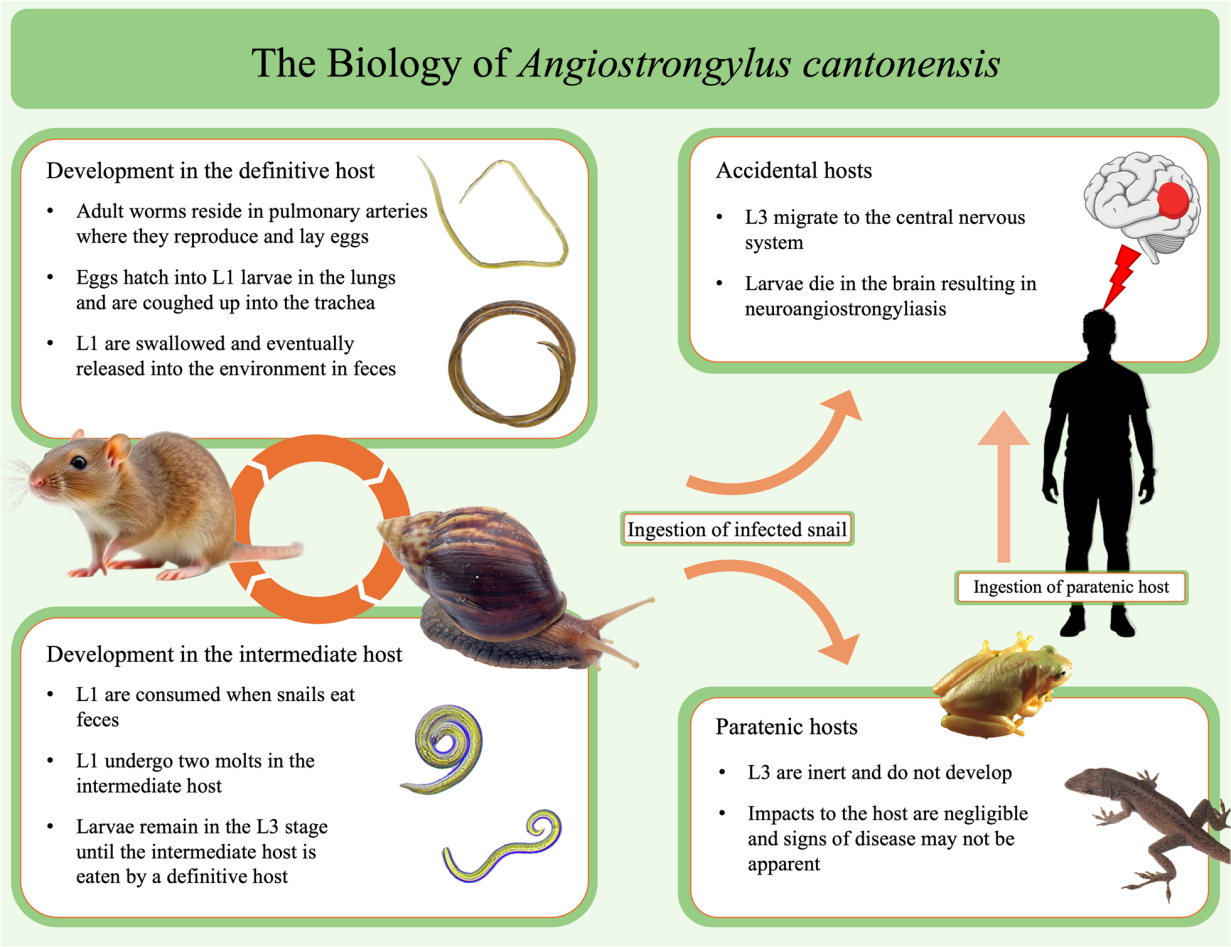
The biology of *Angiostrongylus cantonensis* life stages

*Angiostrongylus cantonensis* (family Angiostrongylidae), the rat lungworm, is a parasitic nematode first described in southern China in 1935 [1]. It has a complex life cycle involving rats as definitive hosts and snails as intermediate hosts (Fig. 1, [2]). *Angiostrongylus cantonensis* adults reside in the pulmonary arteries of rats, where they reproduce sexually and females lay eggs. The eggs travel via the bloodstream to the lungs, where they hatch into first-stage larvae (L1). L1 larvae migrate from the lungs via the trachea into the oropharynx, are swallowed by the host, passed through the alimentary canal, and released in feces. For the life cycle to progress, rat feces must be consumed by snails (or slugs). Once consumed, the L1 molt twice, progressing into the second (L2) and third (L3) larval stages. The L3 become dormant, remaining in the snail host until the life cycle continues when a rat preys on the infected snail. L3 penetrate the rat’s gastrointestinal tract and travel via the circulatory system, eventually entering the brain. In the brain, the L3 undergo two more molts, developing into fourth-stage (L4) larvae and lastly subadult (L5) worms. Young adults then reenter the circulatory system and migrate to the pulmonary arteries and the life cycle begins anew. *Angiostrongylus cantonensis* also has two other types of hosts: paratenic and accidental, neither of which support the entire life cycle. In paratenic hosts, of which there are many [3], L3 larvae do not continue development, but remain infective to other paratenic hosts, accidental hosts, and definitive hosts. In accidental hosts, L3 larvae migrate from the gastrointestinal tract to the central nervous system, notably the brain in humans, where they molt twice, as in the definitive rat hosts, into subadults. However, in accidental hosts, L5 larvae are unable to leave the central nervous system, and as a result cause eosinophilic meningitis—the disease in humans known as neuroangiostrongyliasis, a globally emerging but largely neglected disease that causes diverse symptoms and in severe cases may lead to motor dysfunction, paresis, coma, and sometimes death [2].

## Why study the biology of *A. cantonensis*?

The complexity of the life cycle of *A. cantonensis* and the taxonomic breadth of its hosts make it an excellent model for developmental biology research and for investigating host–parasite relationships. At least 17 species of rats have been identified as definitive hosts of *A. cantonensis* [4], and more than 200 species of intermediate hosts have been reported [5–7]. Turck et al. [3] listed 32 species of paratenic hosts; however, 9 of those were reported as hosts only on the basis of experimental infection. Paratenic hosts of *A. cantonensis* are taxonomically diverse, span both terrestrial and aquatic environments, and include amphibians, centipedes, crustaceans, planarians, and reptiles. Accidental hosts are also taxonomically diverse, including a range of mammals (e.g., humans, non-human primates, dogs, sheep, bats, marsupials) and birds [7]. Given the taxonomic breadth of hosts and the developmental trajectories of *A. cantonensis* within each host type, elucidating the mechanisms underlying larval development would not only enhance understanding of *A. cantonensis* biology, including host compatibility, but possibly that of other parasites with complex life cycles as well.

In addition to its fascinating life cycle, *A. cantonensis* is also an emerging threat to human and animal health as one of the leading causes of eosinophilic meningitis [2]. Eosinophils are important effector cells in the immune response to helminths [8, 9]. In vitro, eosinophils actively destroy helminth parasites [10], particularly during the larval stages [11]. However, in experimental infections of mice and guinea pigs (accidental hosts) with *A. cantonensis*, eosinophils target the subadult stage in the central nervous system, resulting in tissue damage from eosinophil degranulation and its cytotoxic effects [12]. Mechanical damage to brain tissue also occurs from migrating larvae, evidenced by physical lesions along their migratory tracks [13]. In rats, *A. cantonensis* L5 seemingly do not attract eosinophils to the brain because rat eosinophils may lack the receptors needed for recognizing the parasite [12], thus avoiding eosinophil-driven tissue damage. However, with high larval burdens in a rat (> 280 L3), mass bleeding in the meninges can occur, resulting in memory deficits, although most of the severe damage occurs in the heart and lungs [14]. Like many emerging helminthiases, neuroangiostrongyliasis is not well studied, and much remains to be discovered regarding the biology and ecology of *A. cantonensis*.

## The expanding distribution of *A. cantonensis*, an invasive species

*Angiostrongylus cantonensis* is generally considered a tropical/subtropical parasite, limited by low temperatures, and is assumed to have originated in southern China or Southeast Asia, the region in which it was first discovered [2, 7]. It spread rapidly outward into the islands of the Pacific during the 1930s to the 1960s, including to Australia [15, 16] and subsequently to the Caribbean, South America, the southeastern USA, Africa, Indian Ocean islands, the Canary Islands, and the Mediterranean island of Mallorca [7], and most recently to continental Europe [17]. Its range appears to be expanding beyond its previous tropical/subtropical range into more temperate regions, perhaps associated with climate change [18, 19]. The invasive giant African snail (*Lissachatina fulica*) appears to have been a common vector, although rats may have been more important [7, 20]. Human cases of neuroangiostrongyliasis have largely reflected the expanding range of the parasite, with the majority of cases in China, Southeast Asia, and Oceania, but cases have also occurred in Australia, Japan, the Caribbean, and the southeastern USA, and more recently in South America [2, 13], on Indian Ocean islands [21], and possibly in Nigeria [22]. The parasite’s distribution in Africa remains essentially unexplored.

## *Angiostrongylus cantonensis* biology: three advances in the last decade

### The genome

Over the past decade, the genome of *A. cantonensis* has been published three times [23–25], with Xu et al. [24] publishing the most complete genome to date. Analyses of the genomes and predicted proteomes are revealing new insights into not only the mechanisms of *A. cantonensis* parasitism, but also its evolutionary history. Xu et al. [24] identified several gene families that underwent expansion (addition of new genes with similar functions) in the *A. cantonensis* lineage, as well as 454 genes that appeared unique to *A. cantonensis*, most notably genes involved with superoxide dismutase and metallopeptidase activity. Further transcriptomic work by de Mattos Pereira et al. [26] has provided insight into the metabolic pathways of *A. cantonensis*, revealing its ability to synthesize and metabolize major organic macromolecules, including lipids that other parasites such as cestodes and schistosomes are incapable of biosynthesizing [27, 28]. de Mattos Pereira et al. [26] also showed that adult *A. cantonensis* can synthesize serine, cysteine, methionine, and proline, but lack complete pathways for the synthesis of histidine, lysine, phenylalanine, threonine, leucine, isoleucine, and valine—providing insight into host dependency dynamics of the parasite. Although the genome is published, its use in research is still relatively new; thus there remains much more to discover, especially as efforts continue to produce a complete functional annotation of the genome.

### Improved molecular detection assays

Humans and other animals are infected when they eat a raw infected intermediate or paratenic host. The disease is diagnosed via clinical assessment and confirmed definitively when *A. cantonensis* larvae are found in the cerebrospinal fluid (CSF) [29]. Molecular identification of *A. cantonensis* is also performed using quantitative polymerase chain reaction (qPCR) of a patient’s CSF. qPCR assays have typically used primers and probes targeting the internal transcribed spacer region 1 (ITS1), cytochrome oxidase 1 (CO1), or 18S rRNA gene to identify suspected *A. cantonensis* infections, but identification using these markers is challenging [30–32]. ITS1 is not a particularly sensitive marker, often reaching cycle thresholds (Ct) near the limit of detection or being completely negative even though *A. cantonensis* is suspected. 18S primers may generate false-positive results because of sequence similarity with other nematode species [30], and there are instances in which CO1 is unable to distinguish among several nematode species [33, 34]. Sears et al. [35] recently developed a highly sensitive primer/probe assay—AcanR3990—by targeting regions of genomic DNA with tandem repeats. AcanR3990 has consistently lower Ct values (~10 cycles fewer) compared with ITS1, which equates to an approximate 1000-fold increase in sensitivity. Furthermore, AcanR3990 has a relative specificity for *A. cantonensis* of 100%, highlighting its potential to be the qPCR assay of choice. Sears et al. [36] also developed a second primer/probe, RPAcan3990, targeting the same repeated sequences, for use as a field-applicable diagnostic recombinase polymerase assay, bypassing the need for thermocycling conditions used in qPCR. A third assay, Angie-LAMP, was recently developed by Baláž and colleagues [37], also using the AcanR3990 primer, for detecting *Angiostrongylus* DNA in CSF. The method avoids the need for DNA isolation but compares favorably with qPCR methods and could be an ideal assay for rapid field testing of CSF samples.

### Improved laboratory culture methodology

Maintenance of the life cycle of *A. cantonensis* in the laboratory has been undertaken since the 1960s [38], but is demanding because of its complexity. Housing rats and snails requires significant space and is both labor intensive and expensive. In addition to labor and housing costs, both hosts must be killed to retrieve the parasite (with the exception of L1 larvae), which, although performed according to strict and humane regulations, has ethical implications. Efforts to establish development of *A. cantonensis* in culture media external to both the intermediate and definitive hosts have been ongoing since the 1990s [39, 40], but were more recently optimized by Xie et al. [41]. Their methodology allows for in vitro development of *A. cantonensis* eggs into L1 larvae as well as development of L3 into L4 and subadults. However, the method does not allow the complete life cycle to be maintained outside of the hosts, thus not totally eliminating the need for both rats and snails. Nonetheless, this is a crucial step for minimizing the numbers of intermediate hosts needed to obtain worms and reduces the need for specialized animal housing and labor. Furthermore, being able to culture the parasite outside the host opens myriad possibilities for novel research in areas such as parasite development without host influence and stage-dependent drug development.

## Three areas ripe for research

### Host behavior and parasitism

Many parasites alter the behavior of their hosts and these changes may benefit the parasite in some capacity. Perhaps the best known example is that of *Toxoplasma gondii* and its intermediate mouse host whereby it blocks aversion to cat (the definitive host) predator odors [42], thus increasing the probability of transmission. Several studies have explored behavioral responses of intermediate, definitive, and accidental hosts in the *A. cantonensis* host–parasite system [43–46], but none has yielded significant insights into specific host behavioral alterations that could be of adaptive value for the parasite. In trophically transmitted systems, parasites often induce phenotypic changes in their intermediate hosts that alter transmission dynamics via the process of enhancement (increased predation of intermediate hosts), suppression (decreased predation of intermediate hosts), or both (switching) [47]. It is currently unknown how, or whether, *A. cantonensis* uses any of these strategies to complete its life cycle. Considering that it must undergo two molts in the intermediate host to reach the infective L3 stage, which takes approximately 12–16 days [7], suppression should be favored during this time; once L3 is reached, switching to enhancement should be favored. However, this dynamic is complicated if snails eat rat feces more than once across time. This would introduce new L1 into the host, which leads to multiple life stages simultaneously present in the host that could alter the timing of switching. Furthermore, this dynamic could differ across the wide taxonomic range of intermediate hosts because of different host–parasite interactions among snail species. To understand these dynamics better, determining the behaviors of the intermediate hosts, and the underlying mechanisms, is an area ripe for future research. The extent to which *A. cantonensis* alters behavior of rats is also unknown, providing another area for future research. In particular, research should investigate predator–prey interactions of infected rats and snails with one crucial focus on whether rats show any avoidance or preference for infected snails. This has implications for the health and survival of the rat host, which ultimately can impact transmission dynamics.

### *Angiostrongylus cantonensis* neurotropism

The central nervous system is the primary site where L3 continue developing, specifically the brain in humans. While larvae can reach the brain in both accidental and definitive hosts, development only proceeds in the definitive host, and the reasons for this are critically understudied. There are clues, however, about the possible mechanisms. For example, Beaver and Dobson [48] showed high levels of acetylcholinesterase (which catalyzes acetylcholine) activity in *A. cantonensis* larvae in the rat brain, but acetylcholinesterase activity levels are lower in adult worms, which are not in the brain but in the pulmonary arteries. Acetylcholine is primarily synthesized in the brain and is an important neurotransmitter in many organisms, including nematodes [49]. Furthermore, Waymouth’s medium, which was used in the successful cultivation of *A. cantonensis* larvae [39–41], is rich in choline—the precursor to acetylcholine—adding further insight into why the brain is the primary organ of larval development. The brain is also rich in many other important neurotransmitters, which may be needed for *A. cantonensis* development, especially if it lacks the ability to synthesize them. Research into this area would not only provide great insight into the evolution of this fascinating organism and the process/mechanism of development, perhaps of relevance to other parasites with multihost life cycles, but could further improve in vitro cultivation methods as well.

### Consequences of coinfection

Coinfection with multiple pathogens can significantly alter disease progression and outcomes, especially when helminths are involved [50]. Wild rats harbor many types of pathogens concurrently, including bacteria, fungi, viruses, and parasites [51, 52]. There is a substantial gap in knowledge about how coinfections influence the dynamics of *A. cantonensis* in wild rat populations, but there is some evidence that the presence of other helminth species can limit or enhance *A. cantonensis* infection intensity [53]. There is also limited information regarding *A. cantonensis* coinfection dynamics in snails. *Angiostrongylus cantonensis* transmission in some snail species may be impacted by the presence of other helminths. For example, the number of *A. cantonensis* L3 recovered from *Biomphalaria glabrata* also infected with *Echinostoma paraensei* is significantly reduced [54], which lowers the number of larvae that would enter the definitive host. However, that study, while informative, used a snail species (*Biomphalaria glabrata*) that has never been implicated in human neuroangiostrongyliasis, and to the best of our knowledge, has not been recorded as naturally infected with *A. cantonensis* in the wild. Future research into coinfection dynamics should focus on snail species that have been implicated in human eosinophilic meningitis, and are naturally infected, which could lead to targeted methods for combating this emerging infectious disease. The giant African land snail, *Lissachatina fulica*, is an attractive target for snail coinfection research because of its role in human angiostrongyliasis [55], its ability to harbor multiple parasitic helminths [56], and because it is a highly invasive species that has been implicated in the spread of *A. cantonensis* into new regions [57–59]. Furthermore, coinfection studies in both rats and snails could provide new insight into the ecology of *A. cantonensis*, which could aid in disease control efforts. Another consideration regarding coinfections concerns the impact of *A. cantonensis* invasions on native helminths. Interspecific helminth interactions in snails are typically antagonistic [54, 60, 61], often leading to the establishment of only one helminth species in a host. This could have major implications for parasite biodiversity in regions where *A. cantonensis* has recently invaded, especially if it can outcompete native parasites.

## Conclusions

*Angiostrongylus cantonensis* is an intriguing model for research, given its intricate life cycle and its implications for both animal and human health. Understanding the biology of this parasitic nematode not only enriches our knowledge of host–parasite interactions, but also reveals critical insights into its evolution and pathogenicity. With advances in genomics, diagnostic techniques, and laboratory methodologies, the potential to explore new avenues of research is vast, particularly in the realms of host behavior, neurotropism, and coinfection dynamics. As *A. cantonensis* continues to emerge as a significant health threat, the urgency to investigate its complex ecology and biology becomes paramount. By addressing the knowledge gaps surrounding *A. cantonensis*, researchers can develop effective strategies for controlling its transmission and mitigating its impact on public health. Finally, because *A. cantonensis* is an invasive species, understanding potential ecosystem-level impacts, such as biodiversity and food-web impacts, would be of great benefit to conservationists and ecologists alike.

## Data Availability

No datasets were generated or analyzed during the current study.
